# An ecological and digital epidemiology analysis on the role of human behavior on the 2014 Chikungunya outbreak in Martinique

**DOI:** 10.1038/s41598-017-05957-y

**Published:** 2017-07-20

**Authors:** Benjamin Roche, Béatrice Gaillard, Lucas Léger, Renélise Pélagie-Moutenda, Thomas Sochacki, Bernard Cazelles, Martine Ledrans, Alain Blateau, Didier Fontenille, Manuel Etienne, Frédéric Simard, Marcel Salathé, André Yébakima

**Affiliations:** 1 UMI IRD/UPMC 209 UMMISCO, Paris, France; 2 MIVEGEC, Université de Montpellier, IRD, CNRS, Montpellier, France; 3 Centre de Démoustication/Lutte antivectorielle CTM/ARS, Martinique, France; 4 UMR 8197 CNRS/INSERM/ENS IBENS, Paris, France; 5 CIRE Antilles-Guyanes, Fort de France, Martinique, France; 6 School of Life Sciences and School of Computer and Communication Sciences - École polytechnique fédérale de Lausanne, EPFL, Lausanne, Switzerland

## Abstract

Understanding the spatio-temporal dynamics of endemic infections is of critical importance for a deeper understanding of pathogen transmission, and for the design of more efficient public health strategies. However, very few studies in this domain have focused on emerging infections, generating a gap of knowledge that hampers epidemiological response planning. Here, we analyze the case of a Chikungunya outbreak that occurred in Martinique in 2014. Using time series estimates from a network of sentinel practitioners covering the entire island, we first analyze the spatio-temporal dynamics and show that the largest city has served as the epicenter of this epidemic. We further show that the epidemic spread from there through two different propagation waves moving northwards and southwards, probably by individuals moving along the road network. We then develop a mathematical model to explore the drivers of the temporal dynamics of this mosquito-borne virus. Finally, we show that human behavior, inferred by a textual analysis of messages published on the social network Twitter, is required to explain the epidemiological dynamics over time. Overall, our results suggest that human behavior has been a key component of the outbreak propagation, and we argue that such results can lead to more efficient public health strategies specifically targeting the propagation process.

## Introduction

It is well known that for most infectious diseases, transmission intensity fluctuates through space and time^1^. Understanding the spatio-temporal dynamics of infectious diseases provides considerable insights into our understanding of their epidemiology^1–4^ and can help design better strategies for their control^5, 6^. The transmission dynamics of numerous endemic pathogens, ranging from childhood diseases^3^ to vector-borne diseases^7^, have been studied in many different countries^8, 9^. As a result, several factors have been identified that drive the temporal dynamics of pathogen transmission^10^, such as the abundance of vector population for vector-borne diseases^11^, or abiotic factors, which can drive the dynamics of environmentally-transmitted agents like cholera^12^. They also include host behavior, such as the switch between school and holiday periods that shapes seasonality of childhood diseases^13^, or more complex social mechanisms, involving belief-based or prevalence-based feelings^14^.

Local transmission can also be influenced by movements of infectious individuals between cities, especially in small localities and islands^15^. This leads to a spatial structure of pathogen spread which has been extensively documented for many communicable diseases^1, 2, 7^. A pattern of travelling waves from large cities to rural areas has been highlighted for childhood diseases in the UK^3^ or Senegal^16^, as well as for vector-borne diseases such as dengue fever in Thailand^7, 17^.

Although these temporal and spatio-temporal patterns have been documented for many endemic diseases, studies on emerging infections remain rare, creating a large information gap (but see ref. 18 for a recent example). This gap is detrimental to public health because an understanding of the initial stages of propagation is highly relevant for the overall disease epidemiology since it removes the perturbations produced by the appearance of an herd immunity on transmission intensity. Observations during the early stages of an epidemic allow public health authorities to adapt their strategies quickly^19^ and to efficiently organize a control response in the face of such highly unpredictable events. An efficient early response is particularly crucial in the case of emerging infections where most of the population is assumed to be susceptible due to the absence of pre-existing immunity.

The recurrence of these emerging events are currently threatening much of the progress made by public health campaigns during the past decades^20, 21^, making a solid understanding of early-stage epidemic dynamics a clear research priority. To this extent, the temporal and spatio-temporal dynamics of Chikungunya outbreak in Martinique Island represents a unique semi-natural experimental case study. Introduced in December 2013 in Caribbean Islands^22^ and in Martinique^23^, the Chikungunya virus, transmitted by the mosquito *Aedes aegypti* in this area, has spread throughout the whole island, resulting in more than 72,500 infections and 51 deaths^24^ in a population of 400,000 inhabitants. Since the island has a relatively small land area (1,040 km^2^), introductions mainly happen through the largest cities where the harbor and airport are located, allowing us to study the fundamental drivers of the spatio-temporal propagation without the scrambling effect of multiple immigration routes.

We aimed to identify the main drivers of the temporal and spatio-temporal dynamics of the 2014 Chikungunya outbreak in Martinique. We first analyzed the similarity between epidemiological time series recorded within each locality to quantify the propagation through space and time. Focusing on the period preceding a large spatial propagation throughout the island, we then fitted a mathematical model to quantify the contribution of three potential drivers, namely (i) seasonal mosquito abundance (inferred from entomological surveys of infested breeding sites), (ii) awareness of the epidemic in the local human population, and (iii) the interest for protection against the disease (the latter two both inferred from a textual analysis of messages recorded on the social network Twitter) on the temporal dynamics of the outbreak. Based on the identified importance of human behavior during this outbreak, we argue that more research should focus on quantitative assessment of human behavior in the context of emerging infections, and that this component should be considered very seriously in epidemiological response planning in the face of unexpected disease outbreaks.

## Materials and Methods

### Epidemiological Data

Our epidemiological data includes the number of new suspected Chikungunya cases within each locality for each week during the first nine months of the outbreak (from December 2013 to August 2014). Here, we use the number of suspected cases rather than the number of confirmed cases because biological confirmation has not been made routinely after the first 5 months owing to logistic complexity, and thus does not reflect the disease activity over the whole period. Time series of suspected cases is frequently used to estimate epidemiological parameters, especially for infectious diseases with low symptom specificity like influenza viruses^25^. These incidence time series have been estimated based on a network of sentinel practitioners that covers all the localities on the island with at least one medical doctor, resulting in 28 localities considered (6 localities do not have a MD). Each week, the total number of suspected cases collected by sentinel practitioners is extrapolated to the whole island using the ratio between the number of sentinel practitioners that have declared a number of Chikungunya cases (even if it is an absence) over the total number of practitioners in Martinique^26^. These time series have been smoothened through Fast-Fourrier Transform (FFT algorithm as implemented in package “stats” in R software)^27, 28^ where only the dominant frequencies have been kept in order to remove high frequency noise. Population level censuses have been provided by the CIRE Antilles-Guyane (http://invs.santepubliquefrance.fr/Regions-et-territoires/Localisation-et-contacts/Martinique/Cire-Antilles).

### Entomological data

Mosquito abundance is expected to play a significant role in pathogen transmission rate. Taking advantage of long term surveys that have been conducted in Martinique during the past 15 years, we modeled statistically the abundance of mosquitoes through time, assuming that this abundance is linked to the proportion of infested houses visited during routine surveillance, and to the probability of mosquito presence. More precisely, we quantified the contribution of the different weather and land-use data available through Generalized Linear Mixed models^29^ in order to explain the probability of mosquito presence. This step allowed us to derive a robust estimate of the population dynamics of *Aedes aegypti* through time (see details in Supplementary Materials S1).

### Human behavior data

Insecticides are expected to have a limited efficiency according to the high level of mosquito resistance observed in Martinique (more than 80% of mosquitoes show resistance of organophosphate and pyrethroid insecticides through *kdr* mutation within sampled populations)^30^. Therefore, we assumed that the role of human behavior, i.e. through larval source reduction (e.g. removal of stagnant water) and/or individual protection (use of repellents, bed nets, etc.), on pathogen transmission would not have been significantly impacted by these public health interventions. We also assumed that human behavior could be estimated by the amount of relevant messages posted by local residents on the online social network Twitter talking about the outbreak. We quantified the awareness of the epidemic through the number of messages posted on Twitter during the first 9 months of the outbreak that contained the word “Chick*” and “Chik*” (to accommodate potential misspellings of *Chikungunya*). In order to account solely for feelings from people who were located in Martinique during the outbreak, we considered only messages by Twitter users who declared to be based in Martinique. Then, we quantified the protection behavior through the presence of a feeling of protection need expressed in the tweets, which represented a subset of the overall awareness. We textually analyzed the content of each of the 423 tweets messages recorded during this period (including re-tweets) to identify 73 tweets messages with the presence of this feeling (still including re-tweets). Messages classification is detailed in Supplementary Materials (Section S2).

### Spatio-temporal analysis

The spatio-temporal dynamic was first visually assessed by plotting the scaled incidence rate for each locality through time. This visual exploration allowed assessing that the spatio-temporal dynamics could be structured as two separate waves, which we have then tested statistically. Following previous works on the metapopulation of infectious diseases that have identified similar patterns of travelling waves^17, 31^, the similarity between time series was expected to decrease as the geographic distance between them increased^31^. To test the existence of such pattern, we have first quantified the similarity between time series of each locality through (i) the Euclidean distance at each time step of the whole time series and (ii) the differences in the timing of the epidemic peak (the week when the locality has reached its maximal incidence rate). Second, we have quantified the geographic distance between localities as the road length between the centrum of these localities (road network data are available at http://download.geofabrik.de/europe/france/). Finally, we tested for an association between the time series similarity among each pair of locality and their geographic distance.

### Temporal analysis

To quantify the contribution of each of the potential drivers of temporal dynamics throughout the whole island, we used a mathematical model framed within the SEIR framework^32^:

1 $$\frac{dS}{dt}=\mu N-\beta (t)SI-\mu S$$

2 $$\frac{dE}{dt}=\beta (t)SI-{\epsilon }I-\mu E$$

3 $$\frac{dI}{dt}={\epsilon }I-\sigma I-\mu I$$

4 $$\frac{dR}{dt}=\sigma I-\mu R$$

where *S* represented susceptible individuals that are not infected. These individuals could become exposed (*E*) at rate *β*(*t*). After a latency period of average duration 1/ε (asssumed here to be 3 days per individual)^33^, exposed individuals became infectious (*I*) and then can infect susceptible mosquitoes, when mosquitoes could contract the virus from an infectious human, which could subsequently infect other susceptible humans. Finally, infectious individuals recovered at rate *σ* (assumed to be 4 days per individual)^33^ and are then permanently immunized against the disease (*R*). Birth and death rates were identical (*µ*) in order to keep population size constant. Since we focused on an emerging event over a short period of time, we assumed that human demography did not play a significant role in the epidemic dynamics. We included the contribution of the three potential temporal drivers through a forced transmission rate fluctuating through time:

5 $$\beta (t)={x}_{0}({x}_{1}a(t))(1+{x}_{2}p(t+\tau ))(1+{x}_{3}q(t+\tau ))$$

where *x*_*0*_ was the average transmission rate through time, (*x*_1_ *a*(*t*)) represented the impact of mosquito population dynamics on transmission rate (*x*_*1*_ is a constant and *a*(*t*) is the estimated mosquito abundance at time t), (1 + *x*_2_ *p*(*t*)) represented the influence of protection applied by human population, as measured by the textual analysis of tweets (*x*_*2*_ is a constant and *p*(*t*) represents the normalized activity on social network expressing a need for protection) and (1 + *x*_3_ *q*(*t*)) represented the influence of activity – and therefore of the epidemic awareness - as measured on Twitter (*x*_*3*_ is a constant and *q*(*t*) represents the normalized overall activity on Twitter discussing the Chikungunya outbreak). The parameter *τ* represented the lag between the impact quantified on Twitter and its consequences for transmission. We have assumed that the behavior measured on Twitter could be a real-time indicator (*τ* = 0), a delayed indicator (*τ* = 1) when individuals talk on Twitter after having applied the protection, or an anticipated indicator (*τ* = −1) when the Twitter activity reflects the behavior change one month before its impact on mosquito population. The *β*(*t*) function is constrained positive. We then looked for the best estimation of *x*_*1*_, *x*_*2*_ and *x*_*3*_ that allowed the mathematical model to reproduce the epidemiological dynamics observed. Technically, we maximized the likelihood of the model predictions knowing the data by assuming a Gaussian distribution of the model errors through the Nelder-Mead algorithm implemented within the *optim* package in R^28^ (Supplementary Materials S3). Finally, we used a monthly time scale to consider enough tweets to infer human behavior. Therefore, we aggregated all the exploratory variables to this time scale. For each month, we have aggregated each week of epidemiological data (number of cases during weeks that were overlapping across two months have been redistributed to each month according to the respective number of days within each month) and used the mean of vector presence probability during the considered month as a proxy for mosquito abundance.

### Ethics

No individually identifiable medical data has been used in this study.

## Results

**Figure 1:**
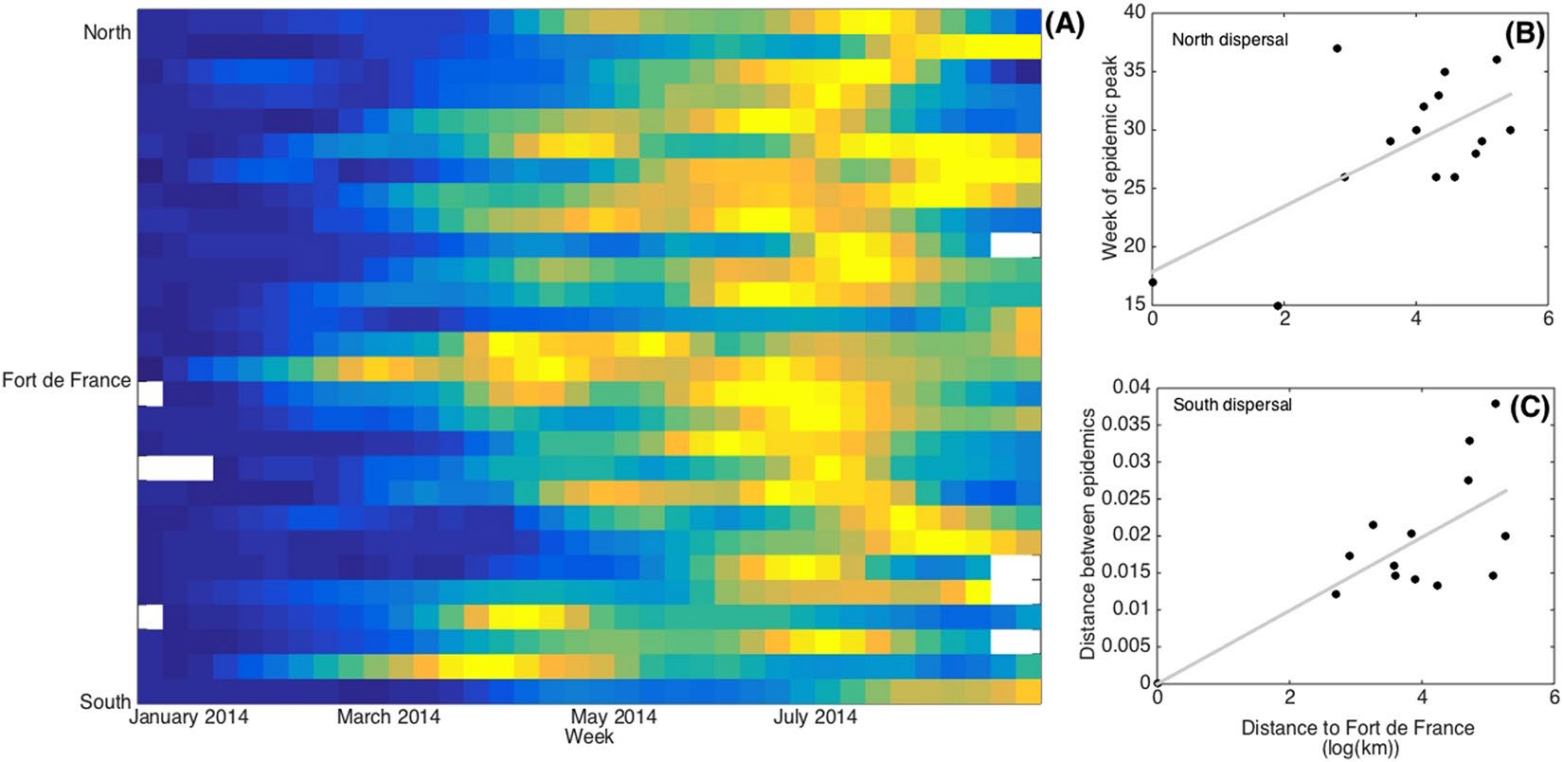
Summary of the spatio-temporal dynamics of the 2014 Chikungunya outbreak in Martinique and its two spatial waves. (**A**) Colors represent the local scaled incidence rate (ranking from 0 in dark blue to 1 in hot yellow) for each locality (rows), ranked from the extreme south to the extreme north of the island according to its position from Fort-De-France, and each week (column). White color represent lack of data. Two waves appear from Fort-De-France, northwards and southwards. Geographic distance (log of km) between different localities through road (x-axis) and (**B**) the week of the epidemic peak (r = 0.6454, p-value = 0.0094) and (**C**) the Euclidean distance between the whole time series (r = 0.72, p-value = 0.0037).

**Figure 2:**
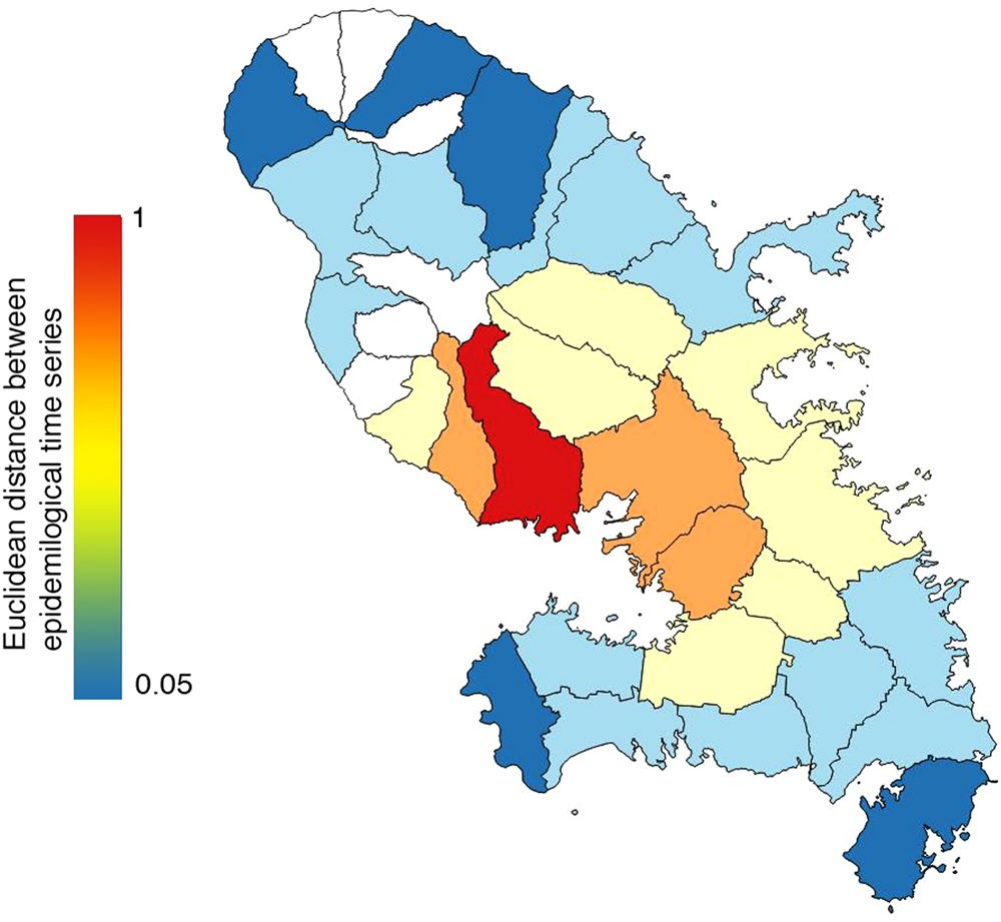
Invasion sequence of the Chikungunya outbreak based on similarity of epidemiological dynamics between each locality and Fort-de-France (similarity has been quantified through Euclidean distance between time series, see main text for more details). Colors follow a gradient from red (considered as epicenter of the epidemics, Fort-de-France, with therefore a correlation of 1) to dark blue (last localities to have been affected, with a correlation close to 0), representing this similarity (localities in white have no epidemiological data). The map has been generated with R software^28^.

The first cases have been declared near to Fort-De-France, the largest city on the Island^34^. The visual analysis of the spatio-temporal dynamics has shown two travelling waves from this city, northwards and southwards (Fig. 1). Interestingly, in the north of the island, the association between local epidemiological dynamics and geographic distance likely relied on the timing of epidemic peak (r = 0.6454, p-value = 0.0094) rather than on the whole series (r = 0.49, p-value = 0.061), while in the south of the island, it likely relied on the whole times series (r = 0.72, p-value = 0.0037) rather than on the epidemic peak (r = 0.39, p-value = 0.16). Based on these correlations, we used the correlation coefficients to characterize the invasion sequence of the virus throughout the island (Fig. 2).

**Figure 3:**
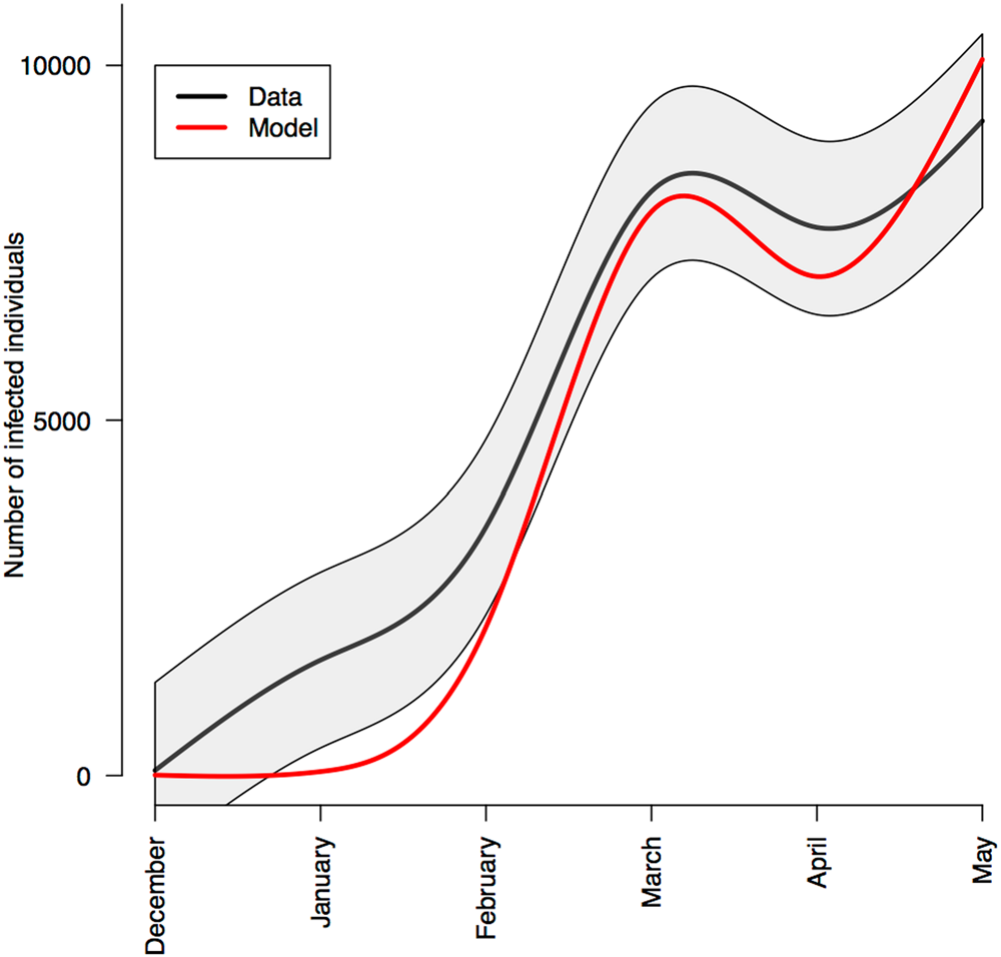
Match between estimated monthly epidemiological data (black, shaded grey area shows the confidence interval based on a Gaussian distribution) and the most parsimonious mathematical model including mosquito abundance and expressed need for protection on Twitter (red line). Estimated transmission parameters are x_0_ = 3.76 10–4, x_1_ = 0.295, x_2_ = 0.644 and τ = −1. See Table 1 for model selection details.

In order to focus on a time period when pathogen dispersal was limited, we limited the analysis of the island temporal dynamics to the time period before it began spreading to remote areas within the island, *i*.*e*. from December 2013 to May 2014. We estimated parameters *x*_*1*_, *x*_*2*_ and *x*_*3*_ for all possible combinations of transmission drivers (mosquito abundance, awareness of the epidemic, and the need for protection) and an anticipated (*τ* = −1), real-time (*τ* = 0) or delayed (*τ* = 1) indicator of human behavior impact on transmission (all the lags are expressed in month). Comparisons between the Mean-Squared Error (using Akaike Information Criterion gives identical results, Supplementary Materials) of each of these models shown that the model including both mosquito abundance and the expressed need for protection, with an anticipated notification on Twitter, explained the best the temporal dynamics observed (Table 1). Moreover, the model predicted around 28,000 new cases over the six-month period, an estimate in line with the slightly more than 30,000 suspected cases recorded by epidemiological data, highlighting the accuracy of this mathematical model despite its simplicity (Fig. 3).

**Table 1:** Results of model estimation.

Parameters included in transmission rate	Twitter as an anticipated indicator (τ = −1)	Twitter as a real-time indicator (τ = 0)	Twitter as a delayed indicator (τ = 1)
None	9088	9088	9088
Mosquito abundance (MA)	6980	6980	6980
Expressed protection need (EPN)	3897	7546	5191
Epidemics awareness (EA)	7797	8240	6878
MA and EPN	2402	4058	3161
MA and EA	7242	8518	5484
EPN and EA	7218	7529	3685
MA, EPN and EA	4389	7639	2675

We show here the squared root of the Mean-Squared Error instead of AIC in order to show the difference between the observed and predicted number of cases. The best model includes the variation in mosquito abundance and the expressed need for protection represented by the proportion of tweets talking about protection against the mosquito in the set of all tweets and retweets that included the word *Chikungunya* (only Twitter accounts declared in Martinique have been considered). The lag period (τ) is expressed in month. AIC values are included in Supplementary Materials.

## Discussion

In this paper, we aimed to identify the main drivers of the Chikungunya outbreak that occurred in Martinique in 2014. We have demonstrated that the temporal dynamics of virus transmission is jointly determined by mosquito abundance as well as by human behavior as inferred from the feelings expressed on the online social network Twitter. We have also shown that the spatio-temporal dynamics of the outbreak follows a travelling wave pattern in two different directions (northwards and southwards).

While mosquito abundance is an expected explanation for the temporal dynamics of the outbreak, the importance of human behavior on the initial stages of an outbreak are less often taken into account, let alone quantified. To the best of our knowledge, this is first time that human behavior has been quantified through social media for a vector-borne disease outbreak, even if social media has been recently used to quantify transmission dynamics of Zika virus^35^. It is consistent with the increasing amount of evidence regarding the role of human behavior in pathogen transmission^14, 36^. Moreover, our analysis suggests that the influence of human behavior is not constant through time, suggesting that communication campaigns have different impacts according to the timing of the operation. This calls for a coordinated outbreak response from public health authorities, municipalities and other partners involved in source reduction, communication campaigns and use of chemical insecticide.

The spatio-temporal spread of the outbreak was shown to follow a two-waves pattern originating from the capital (Fort-De-France), which was the epicenter of the outbreak. Nevertheless, the north and south waves proceeded differently. In the north, where the road network is less dense, only the epidemic peak is associated with distance from Fort-De-France, suggesting that human movement acts as a seed for local epidemics. Conversely, the southward traveling wave, where the road network is more dense, is associated with similarity between the entire time series rather than the epidemics peak. We suggested that the more connected road infrastructure allows for increased movement of infectious individuals that will increase the inter-dependence of pathogen dynamics between localities. While this hypothesis is difficult to demonstrate, we conducted a complementary theoretical approach (Supplementary Materials S4) showing that this explanation could be plausible. The suggestions that the topology of connections between human populations affects the spatial diffusion dynamics of diseases epidemics is in line with results from theoretical studies on the role of network structures on infectious disease spread^37^, especially with vector-borne diseases^38, 39^.

As for any study focusing on epidemiological data, we have made several assumptions that deserve discussion. First, our epidemiological data relies on a sentinel network, and this non-exhaustivity could bring some uncertainty to the validity of our assumptions. Nonetheless, almost 20% of the medical doctors working on the island serve as sentinel doctors, as compared with the 1% of sentinel doctors in metropolitan France^40^, providing support for the relevance and quality of the data we used in our model. Second, we have assumed that these epidemiological data contained the localities where individuals had been infected, although it instead contained the localities where the offices of sentinel doctors are located. Nevertheless, this bias is generally assumed for the study of spatio-temporal dynamics of pathogens and is not expected to play a large role on such analysis^1, 9^. Finally, the low specificity of the Chikungunya symptoms, combined with an unknown number of infected individuals who did not enter the surveillance system for different reasons, can potentially lead to an underestimation of the real incidence. While some cases during the first month of the outbreak may have not been sampled because the Chikungunya virus has never been observed in this area before, which could also explain why our model does not perform well at the beginning of the outbreak, the magnitude of the epidemics (almost 20% of the whole population have been infected) allows us to think that a very intensive sampling effort has been done when the outbreak has been clearly identified. Therefore, this might have a possible quantitative impact on our results but it should not qualitatively change our conclusions.

In this study, we have chosen to use Generalized Linear Models to obtain an average seasonal structure of mosquito population dynamics at the island scale (See Supplementary Materials for more details). Therefore, we did not use the precise mosquito population dynamics of 2014. We made this choice because available entomological data contains a variety of mosquito breeding sites, which are not all the same every year. Therefore, the temporal dynamics over a given season is not accessible because the dynamics estimated is strongly dependent of which breeding sites have been sampled during the considered year. Instead, once compiled over the years, the number and variety of breeding sites was clearly enough to derive a correct average of mosquito seasonal structure through Generalized Linear Models. Moreover, Martinique Island did not record any extreme climatic event that would have serve as seasonal pulse to bring forward or delay the usual mosquito population dynamics. We are therefore confident that the seasonal structure of mosquito population dynamics we have estimated is a reasonable approximation for the year 2014.

The use of feelings expressed on social networks, even if extremely interesting, can potentially introduce a bias since social networks are used only by a small – but rapidly growing - subset of the population^41^. In Martinique during the sampling period, the source of the tweets used in this study is likely to have come from a non-representative population sample, even if more than 74% of the Martinique population has an internet access and social network access is presumably important, as suggested by the 170,000 Facebook subscribers recorded on the island^42^. There are however no good quantitative data to suggest that epidemic awareness and the felt need for protection are very different in different population groups. Previously, the use of such digital epidemiology methods^43, 44^ has been shown to reflect quite correctly the epidemiological dynamics of such emerging pathogens^43^, suggesting that this surrogate can be integrated into mechanistic models involving human host behavior^14^. A careful comparison of this approach against large-scale surveys, if representing the standard approach to infer human sentiments, is lacking right now and should be tackled quickly regarding the potential enormous benefits of having such real-time estimates of human behavior.

Other kinds of analysis could have been also possible. For instance, the spatio-temporal analysis could have been done using statistical model considering time lags^45^. Nevertheless, while this method would allow to quantify precisely the extent of the spatial waves, using such sophisticated technique would be clearly out of the scope of this study which aims identifying the role of human behavior in outbreak propagation. It is worth acknowledging that other hypotheses, not necessarily mutually exclusive, are also possible for this two-waves pattern, such as different socio-economic activities in the two parts of the island that may affect human movements, or several external introductions at different places that may have accelerate pathogen spreading in the South of the island.

Human behavior is increasingly recognized as an important factor for pathogen spreading. The recent example of Ebola outbreaks in Western Africa has seen many dramatic examples of this, underlining how epidemiological response strategies need to consider this component in order to be maximally efficient^46^. While the role of human behavior has long been suggested as a driver for emerging infections^47^, the emergence of online social networks, now widely used throughout the world, opens new opportunities to assess it quantitatively^48^. Our finding that the most parsimonious model for temporal dynamics includes Twitter activity as an anticipated indicator of its impact highlights that analysis of Twitter messages could potentially offer to public health authorities a tool to measure or even predict fluctuations in protective behavior seen in the population. Such quantification, when combined with individuals’ movements and other biotic and abiotic factors known to influence pathogen transmission, can contribute to optimize the efficiency of public health strategies^49^ designed to mitigate the spread of emergent pathogens.

When these results are gathered together, they suggest a strong role of human behavior in the epidemiology of this outbreak. Indeed, our analysis on Twitter is used to be a proxy of human behavior against mosquitoes potentially infected. Similarly, spatial propagation that follows road networks is also suggestive that human movement is driving this dispersion. We therefore believe that our analysis is bringing a quantitative assessment of human behavior in the epidemiology of this outbreak, despite that a clear causal link between human behavior and pathogen transmission is almost impossible to establish, especially for outbreak with this kind of magnitude.

## Electronic supplementary material

Supplementary information
