# Supplementary material for: An ecological and digital epidemiology analysis on the role of human behavior on the 2014 Chikungunya outbreak in Martinique
Source: Sci Rep. 2017 Jul 20;7:5967. doi: 10.1038/s41598-017-05957-y (PMC5519737; doi:10.1038/s41598-017-05957-y)
Supplement: Supplementary file 1 — Supplementary information [file 41598_2017_5957_MOESM1_ESM.pdf]

An ecological and digital epidemiology analysis on the role of human behavior on the 2014 Chikungunya outbreak in Martinique

Supplementary materials

Benjamin Roche[1], Béatrice Gaillard[2], Lucas Léger[2], Renélise Moutenda[3], Thomas Sochacki[1,4], Bernard Cazelles[1,4], Martine Ledrans[5], Alain Blateau[5], Didier Fontenille[2], Manuel Etienne[3], Frédéric Simard[2], Marcel Salathé[6], André Yébakima[3]

1. UMI IRD/UPMC 209 UMMISCO, Paris, France
2. UMR IRD224/CNRS5290/Université de Montpellier MIVEGEC, Montpellier, France
3. Service de Démoustication/Lutte antivectorielle, Fort de France, Martinique, France.
4. UMR CNRS/ENS/INSERM/UPMC IBENS, Paris, France.
5. CIRE Antilles-Guyanes, Fort de France, Martinique, France.
6. School of Life Sciences and School of Computer and Communication Sciences EPFL - Ecole Polytechnique Fédérale Lausanne, Switzerland

S1/ Estimation of *Aedes aegypti* population dynamics

In order to estimate mosquito population dynamics, we analyzed through a logistic Generalized Linear Model (GLM) the presence/absence data of *Aedes aegypti* throughout the Martinique Island thanks to the extensive routine surveillance during the last twenty years (described elsewhere, see (1)). Table S1 shows the results of this GLM.

Fixed effects		
Variable	Coefficient	p-value
**(Intercept)**	-4.81	<2e-16***
**log(Number of breeding sites per household)**	9.649e-1	<2e-16***
**sqrt(Proportion of breeding sites within water tank)**	9.006e-2	<2e-16***
**sqrt(Proportion of breeding sites within flower pots)**	2.368e-2	6.95e-8***
**sqrt(Proportion of breeding sites within small recipient)**	3.593e-2	<2e-16***
**sqrt(Proportion of breeding sites within other places)**	-1.205e-2	0.00638**
**sqrt(Proportion of breeding sites within small plates behind flower pots)**	-2.918e-2	<2e-16***
**sqrt(Proportion of breeding sites within large recipient)**	5.419e-2	<2e-16***
**sqrt(Proportion of breeding sites within tires)**	1.061e-1	<2e-16***
**sqrt(Proportion of breeding sites within trashes)**	1.057e-1	<2e-16***
**sqrt(Distance from locality downtown)**	2.612e-3	<2e-16***
**Average (over the last ten years) of temperature during the previous trimester**	8.677e-2	9.57e-6***
**Rainfall of the current month minus monthly average rainfall during last trimester**	4.96e-4	1.77e-11***
**Average temperature during the last 48H minus average temperature during the last week**	3.363e-2	1.76e-05***
**Random effect**		
**Geographic location**	0.1182	
**Year**	0.1271	
**Month**	0.0497	

Table S1: Generalized Linear Model explaining the presence/absence data of *Aedes aegypiti*. The output variable has been considered as binomial with a logit link function. Only significant variables are shown here. 7197 observations between 2001 and 2014 have been considered.

Through using this GLM, we can therefore extrapolate the population dynamics throughout the island (figure S1), which reflects roughly a sinusoidal function. It is worth pointing out that, in addition to these factors that have been kept, our dataset included also the same set of environmental variables that the one fully

described in (2). These remaining variables were the variables kept after a forward-model selection.

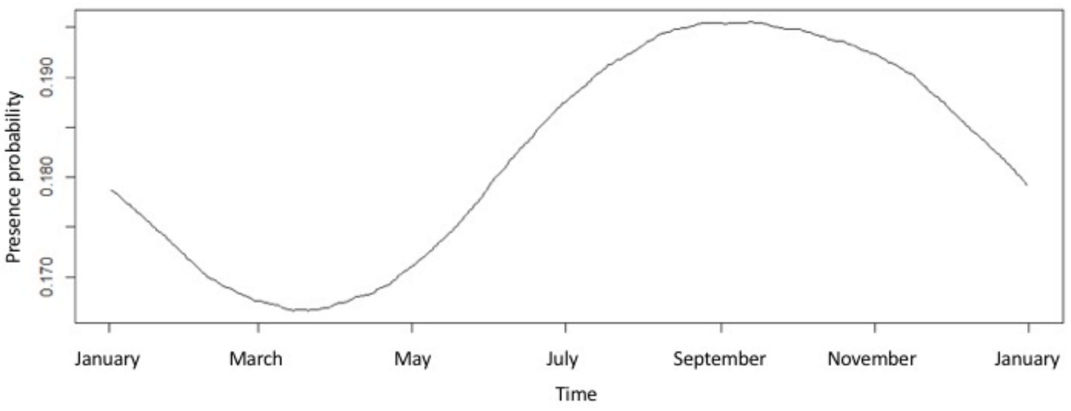

Figure S1: Presence probability through time predicted by the GLM and that is considered in the model fitting detailed in the main text.

It is worth pointing out that the relation between presence of larva in breeding sites and abundance of adult mosquitoes is not simple and probably not linear as we assume. First, there is some delay between the larval and the adult stages. Nevertheless, this delay is about a week for *Aedes aegypti*, while we consider a month time scale in our epidemiological model. Such delay is therefore not able to perturb our results. Moreover, the probability of presence could be not directly proportional to the abundance. However, we consider this dynamic only to identify the trends in mosquito population dynamics, *i.e.,* the periods of high and low abundance, while its quantitative impact on pathogen transmission rate relies on epidemiological parameters that are estimated using the epidemiological dynamics (see Section S3 in Supplementary materials for a full description). We are therefore confident that our estimation of mosquito population is relevant for the purpose of our study.

<u>S2/ Classification of tweets and dynamics through time</u>

We have first identified all the Twitter accounts that have mentioned the word "Chikungunya" during the outbreak in Martinique (from December 2013 to June 2014). Among these accounts, only the ones declared to be located in Martinique in their user profile have been considered. The number of such tweets was interpreted as measure of the awareness of the outbreak.

In order to measure the protection need, two different persons analyzed the content of each message (tweets) three times to identify correctly the tweets expressing protection need. During the first reading, we identified all the keywords associated with protection (the keyword in French is indicated in italics):

Répulsif (repellant), *protéger* (to protect), *anti-moustique* (anti-mosquito), *traitement* (treatment), *raquette* (electric racket used to kill mosquitoes), *huiles essentielles* (essential oils), *prier* (pray), *homéopathie* (homepathy), *vêtement long* (long clothes), *démoustication* (vector control), *précautions* (precaution), *moustiquaire* (mosquito net), *climatisation* (air conditionning), *Dechiktaj* (communication campaign to encourage people removing stagnant water)

Following this, a second reading of those tweets containing any of the above keywords was done once again independently by the two readers to verify their classification as expressing protection need. Finally, a third reading by the two readers was done on the tweets that did *not* contain any of the keywords identified in order to confirm their classification as not expressing protection need.

We note that protection need tweets are a subset of the awareness tweets, as described above. This follows also from the observation that protection need requires awareness of the disease in the first place. Figure S2 shows the dynamics of these two quantities through time.

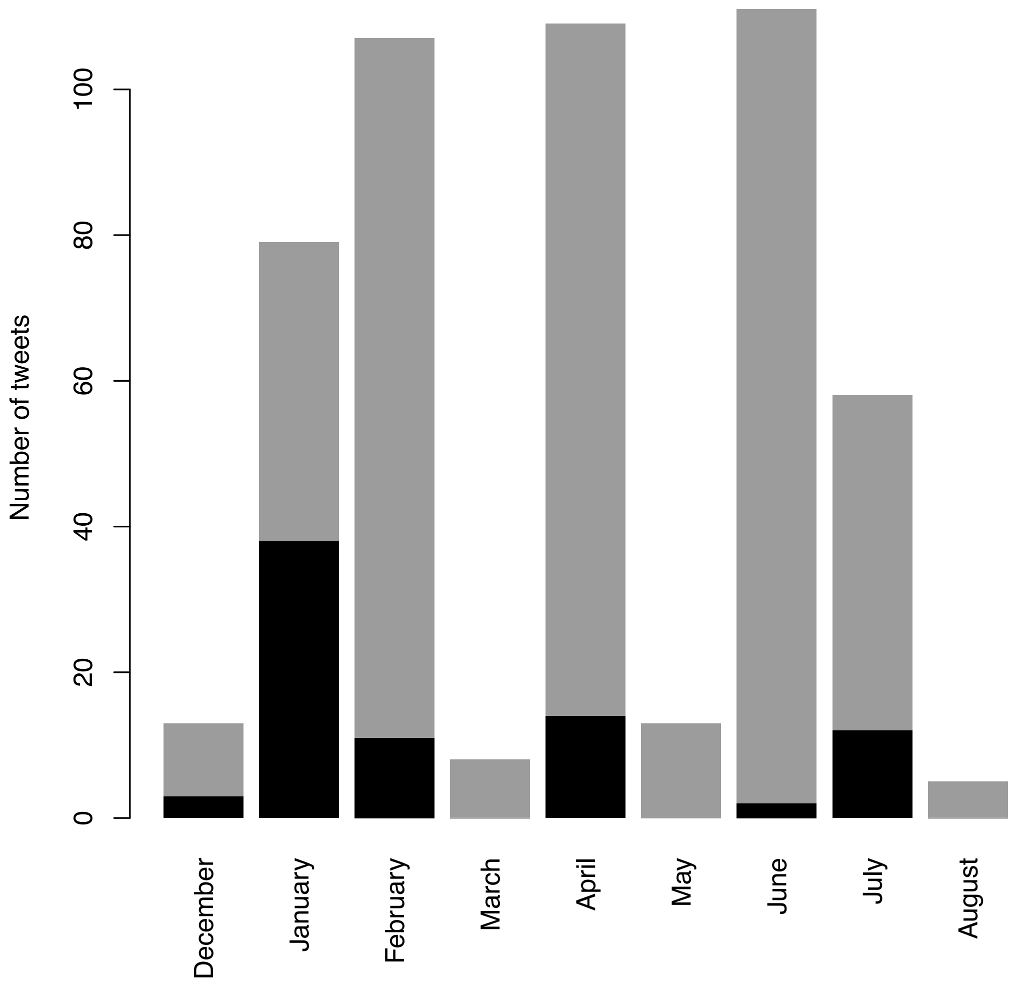

Figure S2: Dynamics of Twitter activity of disease awareness (entire bar) and protection need (black section of bar) during the course of the Chikungunya epidemic.

S3/ Estimation of best parameters

We estimate the likelihood function of our model through the following function (3):

$$L(m(x), \sigma^2 | d) = \prod_i \frac{1}{\sqrt{2\pi\sigma^2}} exp \frac{(d_i - m_i)^2}{2\sigma^2} \qquad (1)$$

where d represents data, m model realizations and σ^2 the variance of the errors. We therefore assume that the errors are normally distributed, as it has been done in similar studies (3, 4).

Then, for each possible set of parameters (combinations of mosquito abundance, epidemic awareness and expressed protection need), we start 50 times the Simplex algorithm to maximize the likelihood with seeds that were randomly uniformly distributed within the range of parameters detailed in table S2. Figures S3, S4 and S5 show the best estimation for all the combinations. It is worth pointing out that most of the seeds were converging to the same parameter values, and the others were converging to parameter values offering a worst fit.

Parameters	Range
**x_0 (mean transmission rate)**	[0.0002 ; 0.05]
**x_1 (contribution of mosquito abundance)**	[0 ; 10]
**x_2 (contribution of epidemics awareness)**	[-10 ; 10]
**x_3 (contribution of protection feeling)**	[-10 ; 10]
**τ (delay in Twitter activity)**	[-1 0 1]

Table S2: Range of seeds explored during the estimation procedure.

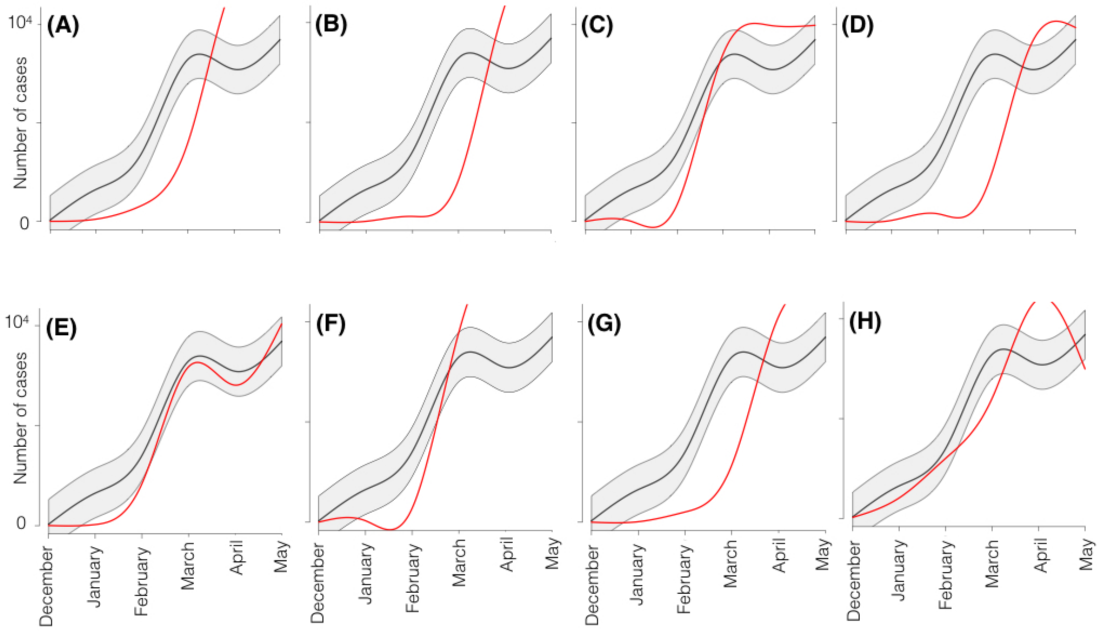

Figure S3: .Best model estimation (with a delay of one month between Twitter activity and the impact on transmission) for the different scenarios: (A) No fluctuations, (B) Only mosquito fluctuation, (C) only feeling for protection need fluctuation, (D) only epidemics awareness fluctuation, (E) combination of mosquito abundance and feeling for protection need fluctuations, (F) combination of mosquito abundance and epidemics awareness fluctuations, (G) combination of protection feeling and epidemics awareness fluctuations and (H) all parameters together.

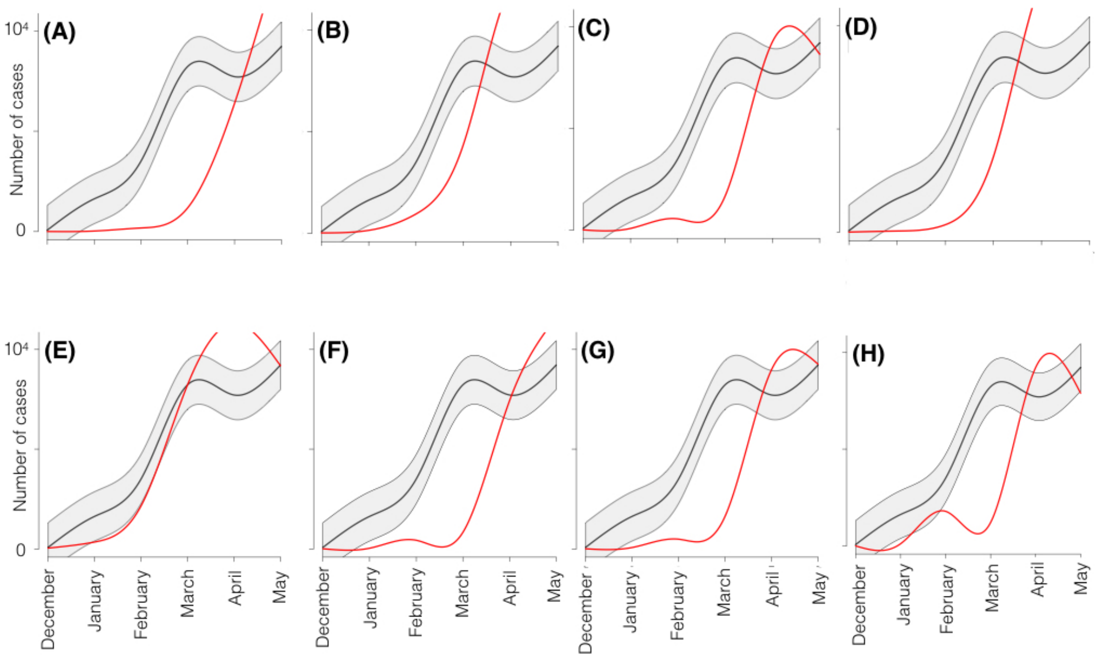

Figure S4: Best model estimations (with no delay between Twitter activity and the impact on transmission) for the different scenarios: (A) No fluctuations, (B) Only mosquito fluctuation, (C) only feeling for protection need fluctuation, (D) only epidemics awareness fluctuation, (E) combination of mosquito abundance and feeling for protection need fluctuations, (F) combination of mosquito abundance and epidemics awareness fluctuations, (G) combination of protection feeling and epidemics awareness fluctuations and (H) all parameters together.

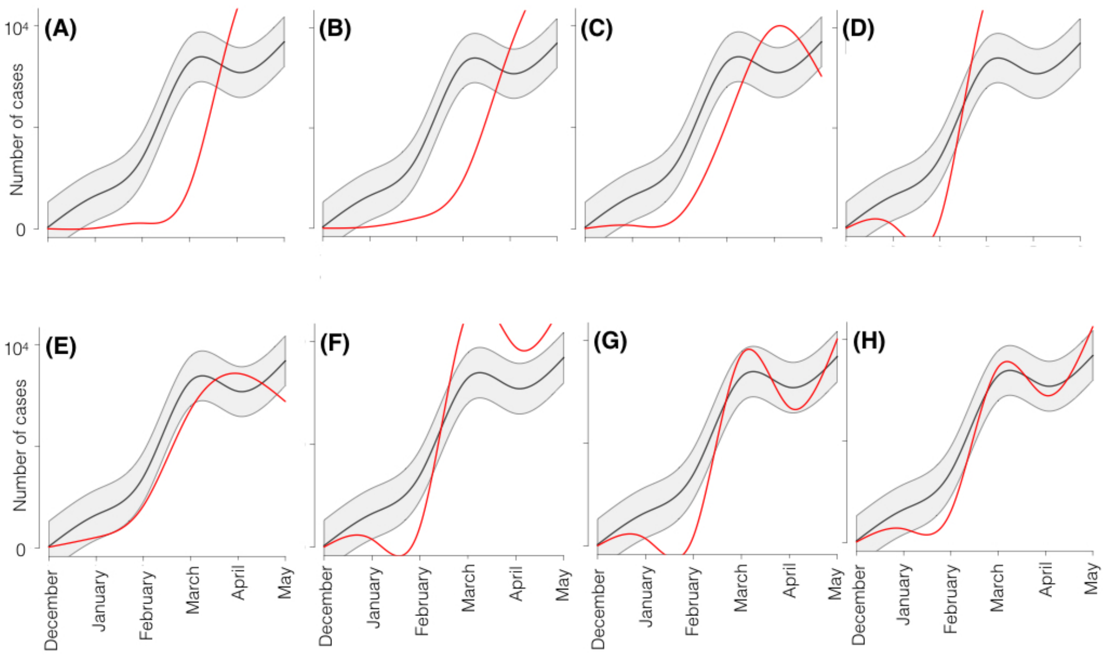

Figure S5: Best model estimations (with an advance of one month between Twitter activity and the impact on transmission) for the different scenarios: (A) No fluctuations, (B) Only mosquito fluctuation, (C) only feeling for protection need fluctuation, (D) only epidemics awareness fluctuation, (E) combination of mosquito abundance and feeling for protection need fluctuations, (F) combination of mosquito abundance and epidemics awareness fluctuations, (G) combination of protection feeling and epidemics awareness fluctuations and (H) all parameters together. These are the best estimations with an advance of one month between Twitter activity and the impact on transmission.

Akaike Information Criterion (AIC) values:

Parameters included in transmission rate	Twitter as an anticipated indicator (τ=-1)	Twitter as a real-time indicator (τ=0)	Twitter as a delayed indicator (τ=1)
**None**	120.52	120.53	120.52
**Mosquito abundance (MA)**	70.79	70.77	70.76
**Expressed protection need (EPN)**	23.99	75.7	25.78
**Epidemics awareness (EA)**	82.18	94.02	59.71
**MA and EPN**	11.52	16.30	14.90
**MA and EA**	66.27	99.80	28.98
**EPN and EA**	65.69	73.35	21.49
**MA, EPN and EA**	18.96	74.12	12.91

Table S3: AIC values for the different models tested.

S4/ Exploration of the role of the road network on the signature of pathogen spatio-temporal dynamics

In the main text, we show that propagation waves are different in the north and in the south of the island (*i.e.,* correlations are significant with the epidemic's peak in the north and with the whole time series in the south). As mentioned in the discussion, one main difference between the north and the south of the island is the road network topology, which is less dense in the north of the island because the presence of the volcano (Figure S6).

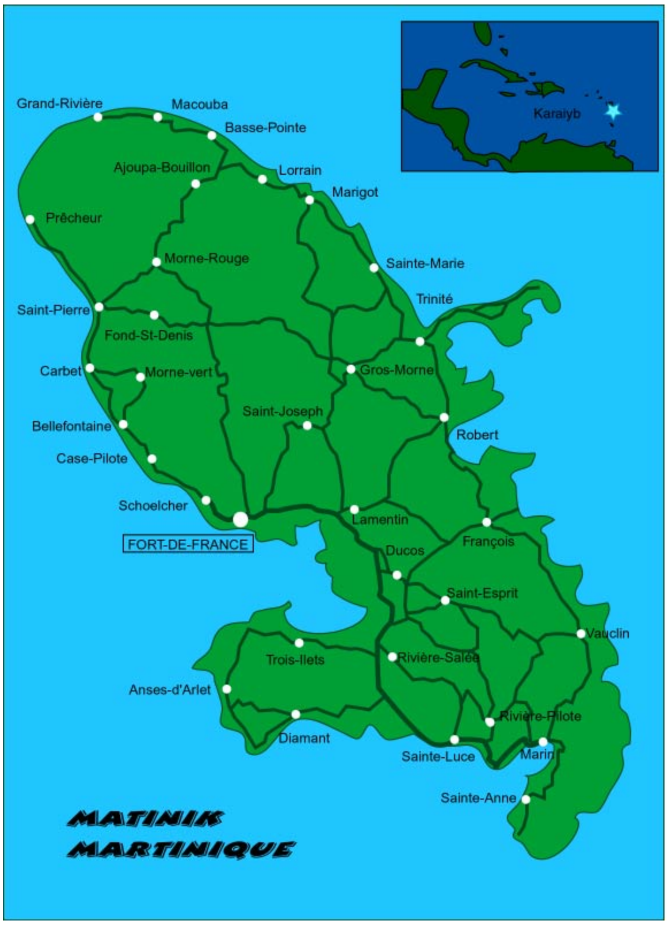

Figure S6: Road map of the Martinique island (OpenCommons license, available at https://fr.wikipedia.org/wiki/Fichier:Carte_de_la_Martinique.jpg)

Here, we want to test the hypothesis that the road network can explain the signature of the observed spatio-temporal dynamics of the outbreak. To do that, we use a simple metapopulation epidemiological model using the following set of ordinary differential equation:

$$\frac{dS_i}{dt} = \mu N_i - \sum_{i=1}^{n}(\beta_{ij}\phi)S_i I_j - \mu S_i \quad (2)$$

$$\frac{dE_i}{dt} = \sum_{i=1}^{n}(\beta_{ij}\phi)S_i I_j - (\epsilon + \mu)E_i \quad (3)$$

$$\frac{dI_i}{dt} = \epsilon E_i - (\mu + \sigma)I \quad (4)$$

$$\frac{dR_i}{dt} = \sigma I_i - \mu I \, (5)$$

where index i indicates the population considered, and n is the number of populations involved. β_{ij} represents the transmission rate from population i to population j. All other parameters are the same as in the main text.

To represent the road network in the north of the island, which is not extremely connected, we assumed a one step matrix as following:

	1	2	3	4	5	6	7
1	β_{ii}	β_{ij}	0	0	0	0	0
2	β_{ij}	β_{ii}	β_{ij}	0	0	0	0
3	0	β_{ij}	β_{ii}	β_{ij}	0	0	0
4	0	0	β_{ij}	β_{ii}	β_{ij}	0	0
5	0	0	0	β_{ij}	β_{ii}	β_{ij}	0
6	0	0	0	0	β_{ij}	β_{ii}	β_{ij}
7	0	0	0	0	0	β_{ij}	β_{ii}

We assumed a transmission rate that is decreasing through the reciprocal distance for the south of the island:

	1	2	3	4	5	6	7
1	β_{ii}	β_{ij}	β_{ij}	β_{ij}	β_{ij}	β_{ij}	β_{ij}
2	β_{ij}	β_{ii}	β_{ij}	β_{ij}	β_{ij}	β_{ij}	β_{ij}
3	β_{ij}	β_{ij}	β_{ii}	β_{ij}	β_{ij}	β_{ij}	β_{ij}
4	β_{ij}	β_{ij}	β_{ij}	β_{ii}	β_{ij}	β_{ij}	β_{ij}
5	β_{ij}	β_{ij}	β_{ij}	β_{ij}	β_{ii}	β_{ij}	β_{ij}
6	β_{ij}	β_{ij}	β_{ij}	β_{ij}	β_{ij}	β_{ii}	β_{ij}
7	β_{ij}	β_{ij}	β_{ij}	β_{ij}	β_{ij}	β_{ij}	β_{ii}

Here β_{ij} represents the inter-population transmission rate and β_{ii} the intra-population transmission rate. Initially, we assume that all β_{ij} are identical among them as well as all β_{ii}among them. Then, we also include a random term (ϕ, which follows a uniform distribution between 0 and 1) in transmission patterns between localities in order to introduce stochastic noise. We have considered seven localities arbitrarily, but the results shown below will remain the same as far as the same assumptions are considered regarding matrix values. Finally, we assume that the population sizes of the different localities decrease linearly with geographic distance from the largest city.

We then wanted to test if the first situation (representing the road network in the north of the Island) and the second one (representing the road network in the south of the Island) can produce the pattern we observe in the data. In the first situation (unidimensional road network, corresponding to the North of the island), the timing of the epidemic peak has a much stronger correlation with geographic distance than the distance between series (Figure S7, A,C,E). However, the Euclidean distance between times series has a much stronger correlation with geographic distance than the timing of the epidemic peak for the second situation (representing the South of island, figure

S7, B,D,F). It is worth mentioning that the gradient in population size is required to observe this pattern.

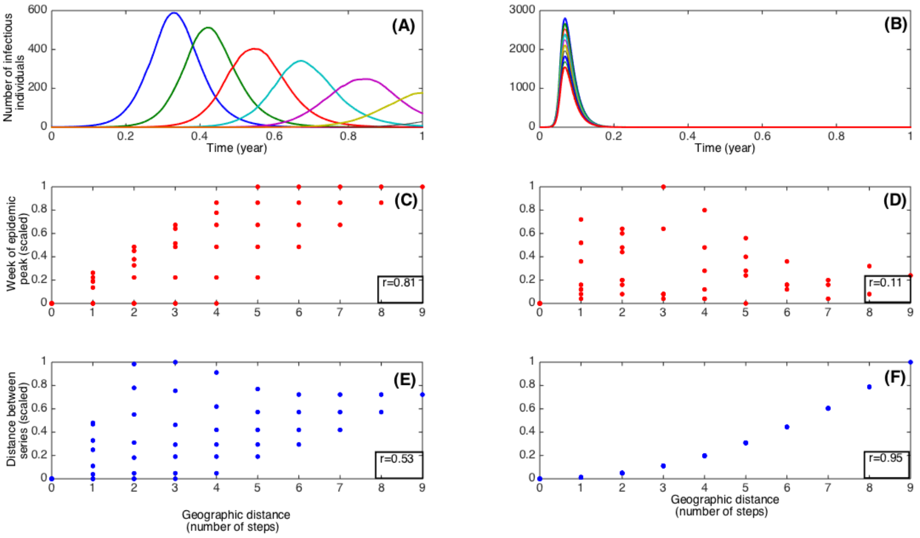

Figure S7: Expected epidemiological patterns with geographic distance if populations are only connected with its neighbors (A,C,E) or with all other populations with a negative relationship with distance (B,D,F). X-axis represent time on panels A and B and geographic distance (number of steps between two localities) on panels C-F. Parameters: $N(i)=(0.66-(i-1)*0.05)*10^4$, $\beta_{ii}=0.03$, $\beta_{ij}=0.003$, $n=10$, $\sigma=7.days.ind^{-1}$, $\varepsilon=4.days.ind^{-1}$, $\mu=80.years.ind^{-1}$.

Bibliography

1. Yebakima A (1990) *Aedes aegypti in Martinique Island*.
2. Roche B et al. (2015) The spread of Aedes albopictus in Metropolitan France: contribution of environmental drivers and human activities and predictions for a near future. *PLoS One*.
3. Choisy M, Guégan J-F, Rohani P (2005) in ed TIbayrenc M (Wiley).
4. Roche B et al. (2009) Water-borne transmission drives avian influenza dynamics in wild birds: the case of the 2005-2006 epidemics in the Camargue area. *Infect Genet Evol* 9:800–805. Available at: http://dx.doi.org/10.1016/j.meegid.2009.04.009.
